# Fear of predation drives stable and differentiated social relationships in guppies

**DOI:** 10.1038/srep41679

**Published:** 2017-02-02

**Authors:** Robert J. P. Heathcote, Safi K. Darden, Daniel W. Franks, Indar W. Ramnarine, Darren P. Croft

**Affiliations:** 1 Centre for Research in Animal Behaviour, College of Life and Environmental Sciences, University of Exeter, Exeter. EX4 4QG, UK; 2 Department of Biology and Department of Computer Science, University of York, York YO10 5DD, UK; 3 Department of Life Sciences, University of the West Indies, St. Augustine, Trinidad and Tobago

## Abstract

Social relationships can have important consequences for fitness in animals. Whilst numerous studies have shown that individuals often join larger groups in response to perceived predation risk (i.e. fear of predation), the importance of predation risk in driving the formation and stability of social relationships within groups has been relatively ignored. We experimentally tested how predation threat influenced fine-scale social network structure using Trinidadian guppies (*Poecilia reticulata*). When perceived predation risk was high, individuals developed stable and more differentiated social ties compared to when perceived risk was low. Intriguingly, social differentiation coincided with shoals being somewhat smaller under high-perceived risk, suggesting a possible conflict between forming stable social relationships and larger social groups. Individuals most at risk of predation (large and bold individuals) showed the most exaggerated responses in several social measures. Taken together, we provide the first experimental evidence that proximate risk of predation can increase the intensity of social relationships and fine-scale social structure in animal populations.

Predators can influence the ecology and evolution of their prey directly by eating them, but also indirectly by influencing the behaviour of survivors^1^. In some cases, the behavioural effects resulting from the fear of predation can actually have stronger and longer-lasting evolutionary consequences for populations compared to direct predation itself^1^,^2^,^3^,^4^,^5^. For instance, risk of predation alone can have significant effects on reproductive rate^6^ and emigration^7^, which may be extreme enough to limit population growth^5^. Many of these population-level effects seem to be driven by the costs associated with trading-off time allocated to foraging and reproductive behaviour against increased vigilance and predator avoidance^1^,^4^. To counter these costs, many prey form social groups or aggregations^8^, where individuals can benefit as a function of group size by, for example, reducing their own predator surveillance effort (the ‘many eyes’ effect^9^,^10^) and reducing their own risk through ‘dilution’^11^,^12^.

The anti-predator benefits of sociality may depend not only on being a member of a group however, but also on the social relationships an individual has with its group members. Whilst most research has focussed on how the strength and stability of social relationships can counter intra-group conflict (mostly in primates^13^,^14^ and birds^15^), with important benefits for foraging success^16^, longevity^17^, and offspring number^18^,^19^, some studies suggest these social relationships might also play a key role in the effectiveness of anti-predatory responses, such as through enhanced reciprocal anti-predatory behaviours or increased cooperation during predator inspections^20^,^21^,^22^. For example, crested macaques (*Macaca nigra*) and dwarf mongooses (*Helogale parvula*) may respond differentially to predator alarm calls depending on their degree of affiliation with the caller^22^,^23^, and several fish species are known to intensify anti-predatory behaviours when associating with preferred social partners^20^,^21^. Perhaps surprisingly however, given the ubiquity of social behaviour in anti-predatory responses^8^ and the importance of social relationships in general (e.g. refs 24 and 25), there have been very few studies on how proximate predation risk drives social differentiation and stable social relationships in animal populations (although see ref. 26 for a recent exception).

We propose that when social relationships are important for anti-predator behaviour, their formation may conflict with the well-established anti-predator benefits of forming larger groups. The maintenance of social relationships often requires individual recognition, which can be cognitively demanding when it involves large numbers of associates such as in the dynamic social interactions typical of fission-fusion societies^27^,^28^. In fact, these cognitive constraints are argued to be the major limiting factor of social group size in primates^29^. Forming stable and differentiated social relationships to enhance anti-predation behaviour may therefore need to be traded off against the formation of large social groups that also benefit individuals under predation risk. The extent to which animals reconcile these potentially conflicting strategies under predation risk is currently unknown.

In this study, we experimentally manipulated perceived risk of predation in replicated mesocosms housing wild-caught Trinidadian guppies (*Poecilia reticulata*) to test the degree to which predation risk alters social differentiation, social stability, and group size. Guppies exhibit fission-fusion social behaviour, where individuals frequently leave and join new shoals over short time frames^30^. Importantly, repeated shoaling interactions between wild fish are driven by social preference^31^ and it is well established that guppies have the cognitive ability for individual recognition based on previous social experiences^32^,^33^,^34^. As with many prey species, guppies frequently inspect their predators^35^,^36^, which involves small numbers of fish leaving the relative safety of a shoal to closely approach and assess the motivation of large predatory fish (e.g. pike cichlids such as *Crenicichla frenata*). Because non-inspecting fish can benefit by observing a predator’s response to inspecting fish, and individuals can reduce their risk during inspection by coordinating their predator approach with others, this behaviour has become a model for studying cooperation^37^. In fact, in guppies, social ties between shoaling fish are a positive predictor of cooperation during predator inspection^21^. These social traits mean that repeated recordings of shoal composition can be used to assess the structure of social relationships (differentiated associations based on social preference (e.g. ref. 38)), and make guppies an ideal species for examining how perception of predation risk drives the fine-scale social structure of animal populations.

When we exposed populations of guppies to cues indicating a high risk from predatory fish^39^, we found, using a social networks approach, that this high perceived risk of predation led to the stabilisation and enhanced differentiation of social relationships compared to control populations. This intensification of social relationships coincided with fish shoaling in smaller groups, which we suggest may reflect a conflict between the anti-predatory benefits of forming larger groups against those of forming stronger relationships.

## Results

### Mesocosm-level effects.

**Figure 1:**
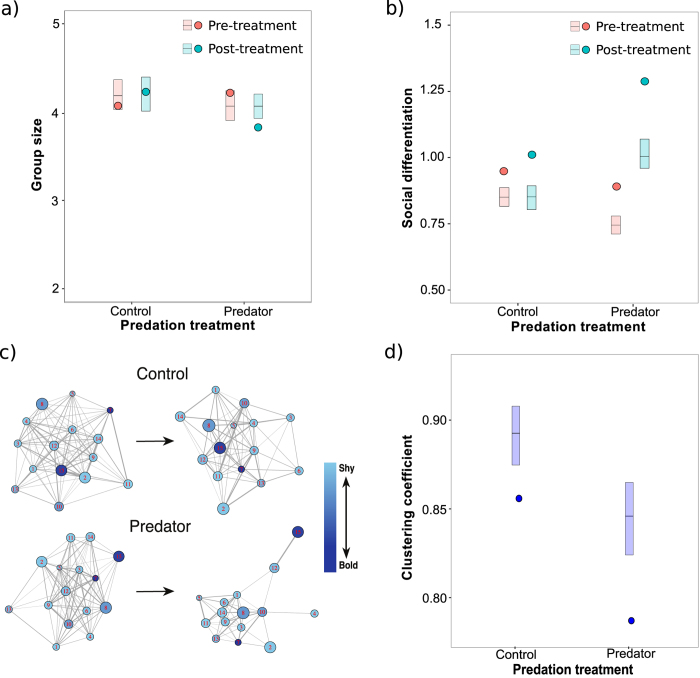
Influence of fear on social behaviour. Patterns of group size (**a**) and social differentiation (coefficient of variation of associations) (**b**) at the mesocosm level across treatments and sampling days. Circles refer to the mean values determined from the imputed (**a**) or observed (**b**) data and rectangles to the 95% confidence intervals determined from permuted data (with the horizontal line within each rectangle illustrating the mean from all permutations). Values represented in (**a**) are from a single randomly chosen imputation and its corresponding 1000 permutations. (**c**) Representative (randomly chosen) social networks created using a spring-layout illustrating the change in social structure between the beginning (left two graphs) and end (right two graphs) of the experiment as a function of experimental treatment. Node number and size refers to the ID and body size of the individual, edge thickness refers to association strength, and each node’s graded colour refers to boldness. (**d**) Observed and simulated mean clustering coefficients from the post-treatment association measures of networks in the two experimental treatments.

Over the 10-day experimental period, mean group sizes in mesocosms became significantly smaller in the predator-exposure treatment (where guppies had been exposed to cues indicating acute risk from predatory fish, see Methods) compared to the controls (treatment x day: P = 0.006; treatment: P = 0.002; day: P = 0.005; Fig. 1a), with post-treatment group sizes being 3.05 ± 0.07 in the predator treatment and 3.48 ± 0.10 (mean ± standard error) in the control. This 12% difference is particularly notable given that group sizes are generally expected to increase in relation to predation risk^8^. Given that the experimental treatment created differences in group size, and group size can influence other social network measures independently of biological effects^40^, we controlled for its effect on further social metrics using permutation techniques (see Methods).

During the experimental period, all 16 experimental populations exhibited significant, non-random social differentiation (measured as the coefficient of variation (CoV) in association strength), showing that fish were forming preferential social ties with specific individuals (Omnibus test; pre-exposure; χ^2^ = 115.40, df = 32, P < 0.001; post-exposure; χ^2^ = 172.44, df = 32, P < 0.001). In addition, risk perception significantly affected the degree of social differentiation, where social ties in the eight populations exposed to the predation cues became more differentiated compared to the eight control populations (linear mixed model (LMM): treatment x day: P < 0.001; treatment: P = 0.006; day: P < 0.001; Fig. 1b and 1c). Differences in social differentiation can be driven by social preferences, but also by environmental influences on spatial behaviour. For example, predation risk could cause individuals to be less exploratory, for instance, by spending more time near refuges and shelters, leading them to associate more frequently with their immediate spatial neighbours and thus increase social differentiation independent of social preferences (e.g. ref. 41). However, we found no evidence that the predation treatment influenced the amount of space used by social dyads during the second sampling period (generalised linear mixed model (GLMM); χ^2^ = 0.27, P = 0.602); indicating that the difference in social differentiation between the two treatments was not driven by variation in space use. In addition, there was no effect of boldness on social differentiation (see Table S1 in Online Supporting information), suggesting that the predation effects on social differentiation we report here were driven by effects on social preference.

Social structure remained consistent in the high-risk treatment (omnibus Mantel test; χ^2^ = 41.31, df = 16, P < 0.001, Mantel r value = 0.13 ± 0.05 (mean ± standard error)), but not for the control treatment (omnibus Mantel test; χ^2^ = 21.44, df = 16, P = 0.164, Mantel r value = 0.07 ± 0.04 (mean ± standard error)), indicating the fish were only maintaining stable social relationships when exposed to predatory cues.

### Individual-level effects

We found that perceived risk of predation influenced the clustering coefficient of individuals. As a local measure (as used here), this metric represents the degree to which an individual’s associates are themselves associated with one another^40^. Coefficients of individuals in the high risk treatment were significantly lower than those in the controls when compared to the permuted values after the experimental period (LMM: P = 0.045; Fig. 1d), reflecting how individuals in the predator treatment were becoming more differentiated in their associations compared to the controls.

**Figure 2:**
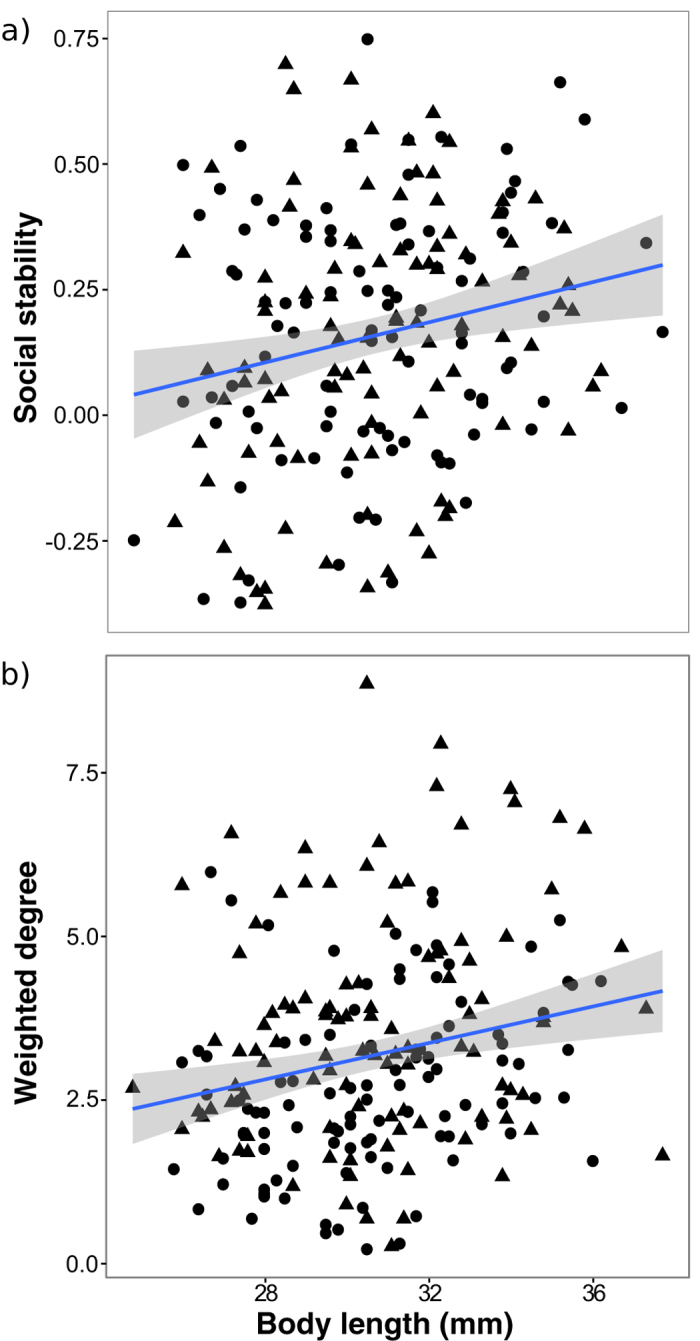
Effect of body size on sociality. Relationship between individual-level social stability (**a**) and weighted degree (**b**) with body size in guppies. Triangles denote fish in the predator treatment, and circles those in the controls. Shaded area around the lines denote the standard errors determined from LMMs on the observed data.

**Figure 3:**
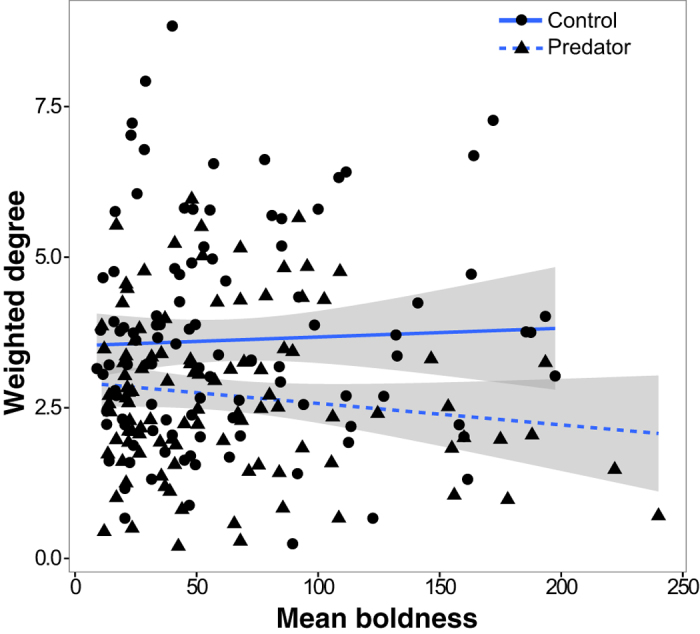
Effect of treatment and boldness on association strength. Interaction LMM plot of the difference between treatments in the effect of mean boldness on weighted degree. ‘Boldness’ refers to the amount of time required for a fish to start moving after a simulated aerial predator attack. Shaded area around each line denotes the standard errors determined from an LMM on the observed data.

The risk of being predated is rarely equal amongst all group members, and larger and bolder fish are known to suffer a greater risk from predators^42^,^43^,^44^. These susceptible individuals may therefore try to offset their increased predation risk using specific social strategies. Furthermore, prey with similar morphological and behavioural characteristics often congregate to reduce predation risk via the oddity effect^45^,^46^,^47^,^48^, a behaviour which may ultimately drive patterns of social heterogeneity and might be particularly likely given that the mesocosms were populated with fish showing a wide distribution of boldness traits (see Methods). We therefore quantified body size and boldness of each individual and assessed how these phenotypic traits related to subsequent social patterns such as social differentiation, group size, and social position as a function of perceived risk (e.g. ref. 41). We found that regardless of experimental treatment, larger fish significantly reduced their clustering coefficients (P = 0.039), had more stable social ties (P = 0.002; Fig. 2a), and had stronger associations (i.e. higher weighted degree) (P = 0.015; Fig. 2b; see Table S1 in Online Supporting information). Bolder fish also formed stronger associations (i.e. higher weighted degree) in the predation treatment but not the control treatment mesocosms (LMM: treatment × boldness interaction; P = 0.028; Fig. 3).

We found no evidence for social assortment by either boldness or body length following the experimental period (boldness predator treatment: χ^2^ = 22.04, df = 16, P = 0.860; boldness control: χ^2^ = 15.41, df = 16, P = 0.510; body length predator treatment: χ^2^ = 15.42, df = 16, P = 0.510; body length control treatment: χ^2^ = 16.00, df = 16, P = 0.540), showing that the social patterns and structures we report here cannot be explained by assortment by these phenotypes.

## Discussion

Social relationships are a hallmark of group living in many taxa, particularly mammals, and yet experimental studies on the environmental factors influencing their development have received relatively little attention^24^. In this study we found that increasing perceived risk from predatory fish caused guppies to form stable and more differentiated social relationships. Interestingly, in contrast to the consensus view that prey should form larger groups under increased predation risk, we found that the enhancement of social relationships under predation risk actually coincided with a slight decrease in average shoal size. To the best of our knowledge, this is the first time that social differentiation and social stability have been experimentally shown to increase as a plastic response to predation threat in any animal.

Humans are one of the few species where social bonds are reported to intensify in dangerous environments, such as between soldiers in active war-zones^49^,^50^ where the strength of these relationships is argued to play an important role in combat effectiveness^49^ and the psychological recovery of veterans^51^. Enhanced social relationships provide many benefits in non-human animals as well^52^,^53^,^54^ and can have significant positive fitness consequences^18^,^19^. As a result, they are likely to be of broad evolutionary importance. For instance, stable and differentiated associations are often considered important for cooperation to evolve as they allow the reciprocation of beneficial behaviours between associating individuals, and thus reduce the potential for exploitation by cheats^55^,^56^. In guppies, the degree to which individuals have previously associated and formed social relationships predicts the likelihood that they will cooperate during risky inspections of aquatic predators^20^,^21^. In the current study we found that predation threat resulted in more stable and differentiated social structures (as also reflected in individuals having lower clustering coefficients in the predation treatment) which may be important in facilitating cooperative anti-predatory behaviours^57^. Corroborating this, we found that larger guppies also had stronger and more stable associations, and bolder individuals in the high predation-risk treatment also formed stronger associations, as would be expected given that these individuals are known to be preferentially targeted by predators^42^,^43^. Given the ubiquity of predator inspection and predator mobbing across many taxa (including fish^58^,^59^, passerine birds^60^, and mammals^61^,^62^,^63^), these results highlight the potential importance of non-lethal predation in the formation of social structures that facilitate cooperation across other species.

The social response to predators may depend on the type of predation risk experienced by prey and the temporal stability of group compositions across different time periods. For instance, a recent study showed that fleeting predatory attacks on great tits by model raptors at artificial feeding stations caused an immediate increase in group composition turnover^26^. Whilst these results seem to contrast with the findings reported here, one possible explanation for the discrepancy may lie in the extent to which cooperative behaviour forms an important component of the antipredator response. Future work examining how different types of predation threat (such as predators that are socially mobbed versus those that are not) influences the stability and differentiation of sociality in prey species provides an exciting area for future research.

Social preference based on individual familiarity forms a major component of shoaling behaviour in freshwater fish^20^,^64^,^65^. Associating with familiar and preferred social partners confers extensive benefits such as enhanced social learning^66^, increased foraging efficiency^67^, and can also facilitate the ability to avoid attacks by predatory fish^20^,^64^. Importantly however, familiarity decreases with increasing group size in guppies and other fish, suggesting that individually recognising multiple shoal members is limited by cognitive constraints^28^,^64^, analogous to how cognitive ability is argued to be the major factor limiting social group size and complexity in primates and other groups^27^,^68^. Our finding that individuals formed stronger social relationships with certain individuals under the threat of predation suggests that in prey species where social relationships have a functional role in anti-predatory behaviour, group size may represent a trade-off between the number of constituent members and relationship quality between those members. This may partially explain the frequent occurrence of small social groups in prey populations that are chronically exposed to high predation risk (e.g. ref. 69).

Taken together, our results provide the first experimental evidence of the key role of perceived predation risk in driving differentiated and stable social relationships in animal societies. Moreover, our results suggest that the well-established anti-predatory benefits obtained from associating with preferred partners may sometimes outweigh those of increased group sizes, providing a novel socially-determined explanation for the variation in group sizes commonly observed in nature.

## Materials and Methods

We used a total of 240 female Trinidadian guppies caught from a 400 m stretch of the Aripo River in the Northern Mountain Range of Trinidad (N10°40 W61°14´), characterised as a high-predation risk location due to the presence of major guppy predators such as the pike cichlid *C. frenata*^69^. Guppies were caught over two collection trips four days apart (120 females per collection), allowing us to stagger the data collection and run 4 batches of 4 mesocosms (see details below). We used females in our study because guppies often form sex-assortative shoals in the wild, with female guppies naturally forming stable same-sex groups compared to males that associate disassortatively (to enhance mating opportunities)^70^. Guppies from each collection were equally allocated between two aquaria (76Lx46Wx46 H cm and a water depth of 35 cm) in a laboratory maintained at 24 °C with a 12:12 light:dark cycle.

After allowing fish to settle for 48–72 hours (balanced across treatments), we then recorded their individual boldness response to a simulated aerial predator. Guppies respond to attacks by aerial predators such as green kingfishers (*Chloroceryle americana*) by darting to the river bottom and remaining motionless. The time taken for fish to start moving again differs repeatedly between individuals^71^. We assayed each fish’s boldness twice: first after acclimatisation (day two-three post-capture), and after the experiment finished (day 14–15). Each female was tested separately in a 46Lx31Wx30 H cm aquarium, which had three sides covered with opaque material, and had a water depth of 15 cm. To reduce stress, a separate shoal of female guppies (sampled from fish not used in the experiment and habituated to the simulated aerial predation strike) was placed in a perforated, transparent cylinder (10 cm radius) on one side of the test aquarium, and all fish were then habituated to the test tank for five minutes. To simulate the aerial predation strike, an 11 mm diameter metal nut attached to a monofilament line (preventing it from striking the bottom of the tank) was dropped into the centre of the test aquarium from a height of 33 cm using a remote pulley system. Boldness scores were determined by the amount of time taken for the focal fish to move following the freezing response. Boldness scores showed significant within-versus-between repeatability after square-root transformation (r = 0.206; ANOVA on within-ID scores: F_1,220_ = 15.63; P < 0.001), validating their use as a personality trait^72^.

To create the experimental populations, fish from each test day were ranked by their boldness scores and allocated systematically to populations (with 15 fish in each population), so that each population contained fish with a similar distribution of boldness scores. This process was repeated for each collection and batch of testing to give a total of 16 populations. Since guppies are known to form non-random associations with other individuals based on their behavioural phenotypes (e.g. ref. 73), this setup ensured that individuals had similar opportunity to choose their social partners based on this trait across all experimental populations. Before being allocated to the experimental populations (after boldness testing), fish were individually tagged using visual implant elastomer (see ref. 74 for details), which has previously been shown to have no effect on shoaling behaviour in guppies^30^, and measured for total length, and then placed in recovery tanks for 24 hours with API STRESS COAT®. Each population was then placed in a separate outdoor circular pool (‘mesocosm’) 180 cm in diameter and 15 cm deep, and allowed to acclimatise for 24 hours. The mesocosms were housed outdoors under natural field conditions at the University of the West Indies campus in St Augustine, Trinidad and Tobago. Substrate added to each mesocosm that originated from the same river that the fish were collected from provided the majority of food (mostly naturally growing algae), although each mesocosm was also supplemented with commercial fish flakes fed twice a day *ad libitum*.

### Predation treatment

For the next twelve days, mesocosms were randomly assigned to either a predation or control experimental treatment (n = 8 predation and n = 8 control), ensuring that treatments were not spatially clustered. In the predation treatment, guppies were exposed to a model pike cichlid (a 13 cm long fishing lure with similar shape and colour to *C. frenata,* deployed into the mesocosms using a pole) for 10 minutes each day, accompanied by conspecific olfactory alarm cues (released during predator-induced mechanical damage to the dermis^75^) sprayed directly onto the water above the model. Olfactory alarm cues were collected from conspecific donors following anaesthesia, cervical dislocation, removal of internal organs and tail fin, homogenisation of the remaining tissue, and filtering through polyester filter floss with 500 ml of aged tap water. 10 ml of the resultant liquid was then sprayed onto the water’s surface. In the control treatment, mesocosms were sprayed with 10 ml of aged tap water only and the water was disturbed in a similar manner to when the model predatory fish was introduced in the predator treatment (i.e. the water was disturbed with an identical pole used to deploy the model pike cichlid).

### Association patterns

We quantified associations using the ‘gambit of the group’ approach^76^,^77^ on day two and day 12 after fish were released into the mesocosms. Sampling was done by two observers, allowing one mesocosm from each treatment to be sampled simultaneously to control for time of day across treatments. Each mesocosm was divided into four ‘quadrants’, and the observer took photographs of all social groups within that quadrant every minute for 15 minutes, before moving onto the next until the entire mesocosm had been sampled. Shoal membership was defined as individuals within four body lengths of one another using a chain rule (i.e. shoal membership was assigned to all individuals that were connected to at least one other that matched this criteria)^78^. All photographs were taken from ~90 cm away using a Nikon D40x digital SLR camera.

### Statistical analysis

Association matrices were calculated separately for each sampling period using the simple ratio index (SRI), which calculates the proportion of times that two individuals were seen associating with one another as a function of the total number of times they were seen^79^.

### Mesocosm-level analysis

We investigated whether the predation treatment influenced the population level shoaling patterns and global properties of the networks, specifically mean group size, social differentiation, and social stability, and secondly whether individual phenotypic factors affected these social patterns. Between the first and second association sampling periods, one fish from each of five mesocosms was lost (one fish from a control and one from each of four predation treatment mesocosms). The cause of this loss was uncertain, but could have been due to bird predation (no dead fish were recovered). Behavioural responses towards avian predators are fundamentally different to those towards predatory fish, most notably in that the former do not involve the complex social behaviours associated with the latter such as cooperative predator inspection, and in fact there is very little evidence for social behaviour playing an important role in avoiding avian predators at all in this species^69^. We nevertheless tested for any influences that this fish loss could have had on social behaviour (see below), and also controlled for any influences the resulting differences in population size could have on group sizes using imputation techniques. Our imputation method specifically controlled for any effects of fish loss on population (and thus group) size by removing single, randomly selected fish from each of three randomly selected control treatment mesocosms (to standardise the population sizes between treatments that differed due to fish loss). After creating the imputed dataset, we ran a GLMM with mean group size in each mesocosm as the response variable, and included day, experimental treatment, and their interaction as covariates. The distribution of each model’s coefficient was then compared against a distribution of 1000 randomised coefficients, where each GLMM had been run on a permuted dataset where group sizes had been shuffled between days and within mesocosms 1000 times. This entire procedure (GLMM coefficients based on imputed data compared to 1000 GLMM coefficients based on permuted data) was then run 500 times, and comparing our imputed coefficients against the distributions of null coefficients allowed us to obtain the overall P value.

We ran an LMM with the group sizes an individual was a member of as a response variable and that individual’s coefficient of variation as a covariate (both using the post-treatment exposure association scores), including individual ID and mesocosm as random effects, which showed a significant inverse correlation (χ^2^ = 15.51; P < 0.001). Given this, we controlled for group size effects on further social metrics using permutation techniques (see below).

We determined if guppies were associating non-randomly by comparing the global network coefficient of variance (CoV) of the SRI for both sampling periods of all networks against null distributions of randomised coefficients. Null coefficient distributions were created by swapping two randomly selected individuals from two groups within a mesocosm 2000 times (each time randomly reselecting individuals) before recalculating the coefficient of variation, and then repeating this entire process to obtain 10,000 random coefficients. This method retains and thus controls for important aspects of the network, such as group size and number of observations for each individual fish^80^.

Because heterogeneity in social associations could be driven by individual differences in space use^41^, we also wanted to determine whether perceived risk of predation could be influencing the spatial distribution of social relationships of guppies (for instance, by making them less exploratory or active, or by causing their movements to be restricted to nearby refuges that they shared with other individuals), which might contribute to differences in social differentiation unrelated to social preference. Using data from the post-treatment sampling period, we ran a Poisson GLMM where the number of quadrants visited by a particular dyad within a mesocosm was used as a response variable, predator treatment included as a predictor, and fish ID (within the dyad) and mesocosm included as random effects.

To compare the effect of the experimental treatment on social differentiation, we calculated the CoV for each mesocosm’s association matrix for each sampling day. This was then used as the response variable in a LMM with mesocosm as a random effect, and day and predation treatment with their interaction as predictors. We also included the number of fish lost within a mesocosm as a covariate to test for and control for any influences this may have had on the social differentiation. Statistical significance of each term was determined by comparing the coefficients from the observed data against those of a null coefficient distribution, created by running LMMs on randomised data created using the same permutation method detailed above when testing for non-random associations based on the CoV. After showing a non-significant effect of fish loss (P = 0.076) this term was dropped from the model. Our best models were determined, in all cases that use permutations, using a stepwise backwards-elimination procedure, where non-significant terms (determined using the permutation tests) were removed (with non-significant term’s statistical details being reported at the point prior to their removal).

The social stability of each network was determined using Mantel tests, using the R package ‘ecodist’^81^, to correlate the association matrix on the first observation day with the second observation day (after the 10-day experimental treatment period) using 10,000 permutations to obtain a P-value (again, using the same permutation method as detailed above). The overall stability for the two treatments was then obtained by combining the mesocosm P-values within the two treatments using Fisher’s Omnibus test^82^.

We determined whether heterogeneity in social relationships was driven by assortativity by similar phenotypes (either by boldness or by body length, both of which are known to occur in guppies^73^,^74^) by calculating Newman’s assortativity coefficient for associations in each mesocosm from the last sampling period (taking into account weighted edges with the R package ‘assortnet’^83^). To obtain the significance of this assortativity, each mesocosm’s coefficient was compared to a distribution of 10,000 randomly generated coefficients, again using the same permutation process detailed above (where each randomised coefficient was created by swapping two individuals 2000 times). Overall assortativity for both experimental treatments was then calculated using Fisher’s omnibus test^82^.

### Individual level analysis

We calculated four social network metrics (using the R package ‘igraph’^84^) for each individual: its degree (the number of individuals it associated with), weighted degree (the overall strength of its social associations), betweenness-centrality (the total number of shortest paths between any pair of individuals that pass through the focal individual), and clustering coefficient (the degree to which an individual’s associates are connected to one another). Degree, weighted degree and betweenness-centrality are good indicators of an individual’s centrality within a network that are known to be repeatable in several species^85^,^86^. Centrality can have important implications for how information, such as that pertaining to predation risk, could be transmitted through a network. We also used clustering coefficient because it quantifies social cohesion, which we predicted *a priori* would be affected by our treatment^40^. We ran LMMs to determine if phenotypic factors predicted mean group size, social stability, social differentiation and our four social network metrics at the individual level. We also ran analyses on ‘change’ for mean group size, social differentiation and the four social network analyses, creating the ‘change’ variable by subtracting the values for the first from the second. In all models, we included boldness, body length, predation treatment and their interactions as predictors, and mesocosm as a random effect.

In the individual group size analysis, the size of each group an individual was a member of during the second observational period was used as a response variable (the second day was used so that we could determine the effect of our experimental treatment, which had not begun by the time the first association patterns had been recorded). We determined each term’s significance using a permutation test where the coefficients for each predictor were compared against a null distribution of coefficients, calculated by shuffling group sizes between individuals within each mesocosm and re-running the LMM. We also ran a separate GLMM to address whether individual social differentiation was related to group size. Here, group size was used as the response variable, social differentiation as the predictor (both social differentiation and group size being based on the post-treatment association measures), and mesocosm being included as a random effect. Social differentiation for each individual was calculated as the CoV of its SRI scores obtained from the second observation period.

Each individual’s social stability was calculated by running a Pearson’s correlation on its SRI scores (i.e. the SRI scores with the other 14 fish in that mesocosm) from the first observation period and the second observation period. The Pearson’s correlation coefficient for each individual was then used as a proxy for social stability.

We determined the effect of the experimental treatment and other phenotypic factors on social position over and above the effects on group size. To do this, we used a permutation approach that maintained group size distributions to obtain P-values for the models on group- and individual-level CoV, individual social stability, individual social differentiation and the different social network metrics. Specifically, P-values were obtained by comparing observed-data coefficients against a randomised distribution of coefficients. Randomised coefficients were generated in a similar way to the global CoV described above, that is by randomly selecting two groups within the same day and mesocosm and swapping one individual from each (two individuals in total) between these groups, repeating this 2000 times within each permutation, and then running 10,000 permutations in total to generate the randomised social metrics^80^. This procedure ensured that the distribution of group sizes in all permutations remained the same, and also that each individual was observed the same number of times.

Between the final association recordings and the final boldness screenings (two days apart), a further 13 guppies disappeared (seven from the predation treatment mesocosms and six from the control mesocosms; a total of 7% mortality rate throughout the experiment). There was no significant difference in mortality rate between experimental treatments (Chi squared test: χ^2^ = 0.11, P = 0.738). Importantly, any further influence of population size on our response variables were controlled for in the permutation tests. All mixed models were done using ‘lme4’^87^ in R^88^.

### Ethics Statement

The procedures outlined in this study were conducted in accordance with the ASAB/ABS Guidelines for the Use of Animals in Research.

## Additional Information

**How to cite this article**: Heathcote, R. J. P. *et al*. Fear of predation drives stable and differentiated social relationships in guppies. *Sci. Rep.* **7**, 41679; doi: 10.1038/srep41679 (2017).

**Publisher's note:** Springer Nature remains neutral with regard to jurisdictional claims in published maps and institutional affiliations.

## Supplementary Material

Supplementary Information
