# Supplementary material for: Fear of predation drives stable and differentiated social relationships in guppies
Source: Sci Rep. 2017 Feb 2;7:41679. doi: 10.1038/srep41679 (PMC5288725; doi:10.1038/srep41679)
Supplement: Supplementary Information [file srep41679-s1.doc]

**Fear of predation drives stable and differentiated social relationships in guppies**

Robert J. P. Heathcote; Safi K. Darden; Daniel W. Franks; Indar W. Ramnarine & Darren P. Croft

We ran two linear models to determine whether there was a difference in boldness scores between the treatments. We log-transformed the boldness scores (to conform to a Guassian distribution) for both the pre- and post-experimental boldness assays and used these as the response variables in each. The sole predictor in each was the experimental treatment. Results from both models showed no difference in boldness between the experimental treatment (Pre-experimental boldness: F_1,216_=0.03, P=0.871; post-experimental boldness: F_1,207_=0.50, P=0.482).

Table S1: Statistical summary of factors that predict an individual guppy’s social network metrics. Significant terms are included in bold. The results for non-

| **Response variable** | **Predictors** | **Coefficient** | **P value** |
| --- | --- | --- | --- |
| Binary degree change | Treatment×boldness×length | 0.0040 | 0.472 |
|  | Boldness×length | -0.0039 | 0.198 |
|  | Treatment×length | 0.0112 | 0.314 |
|  | Treatment×boldness | 0.0202 | 0.415 |
|  | Boldness | -0.0007 | 0.137 |
|  | Length | 0.1412 | 0.384 |
|  | Treatment | 2.0987 | 0.471 |
| Weighted degree change | Treatment×boldness×length | 0.0012 | 0.498 |
|  | Treatment×length | -0.0062 | 0.258 |
|  | Boldness×length | -0.0016 | 0.153 |
|  | Treatment×boldness | 0.0077 | 0.198 |
|  | Boldness | -0.0004 | 0.463 |
|  | Length | 0.0369 | 0.206 |
|  | **Treatment** | **1.1937** | **0.029** |
| Clustering coefficient change | Treatment×boldness×length | -0.0025 | 0.616 |
|  | Treatment×length | 0.0314 | 0.674 |
|  | Boldness×length | 0.0012 | 0.195 |
|  | Treatment×boldness | -0.0005 | 0.123 |
|  | Boldness | 0.0002 | 0.399 |
|  | **Length** | **-0.0870** | **0.039** |
|  | **Treatment** | **1.2361** | **0.046** |
| Final binary degree | Treatment×boldness×length | 0.0003 | 0.296 |
|  | Treatment×length | -0.0757 | 0.371 |
|  | Boldness×length | -0.0014 | 0.372 |
|  | Treatment×boldness | -0.0034 | 0.373 |
|  | Boldness | -0.0021 | 0.053 |
|  | Length | 0.1088 | 0.079 |
|  | Treatment | 1.4590 | 0.106 |
| Final weighted degree | Treatment×boldness×length | 0.0015 | 0.460 |
|  | Treatment×length | -0.0464 | 0.293 |
|  | Boldness×length | -0.0006 | 0.265 |
|  | **Treatment×boldness** | **0.0042** | **0.028** |
|  | Boldness | 0.0008 | 0.055 |
|  | **Length** | **0.0641** | **0.015** |
|  | **Treatment** | **0.8509** | **<0.001** |
| Final clustering coefficient | Treatment×boldness×length | -0.0002 | 0.352 |
|  | Treatment×length | 0.0068 | 0.387 |
|  | Boldness×length | 0.0001 | 0.448 |
|  | Treatment×boldness | -0.0002 | 0.401 |
|  | Boldness | 0.0001 | 0.495 |
|  | Length | -0.0058 | 0.093 |
|  | **Treatment** | **0.0688** | **0.045** |

significant terms are included at the point of their deletion from the models.

Table S2: Statistical summary of factors that predict a guppy’s individual social stability, group size and social differentiation. Significant terms are included in bold. The results for non-significant terms are included at the point of their deletion from the models.

| **Response variable** | **Predictors** | | **Coefficient** | | **P value** | |
| --- | --- | --- | --- | --- | --- | --- |
| Individual social stability | Treatment×boldness×length | -0.0001 | | 0.455 | |  |
|  | Treatment×boldness | 0.0001 | | 0.459 | |  |
|  | Treatment×length | 0.0119 | | 0.135 | |  |
|  | Boldness×length | 0.0002 | | 0.118 | |  |
|  | Treatment | -0.0293 | | 0.241 | |  |
|  | Boldness | -0.0003 | | 0.273 | |  |
|  | **Length** | **0.0194** | | **0.002** | |  |
| Individual group size | Treatment×boldness×length | 0.0004 | | 0.217 | |  |
|  | Treatment×boldness | 0.0016 | | 0.493 | |  |
|  | Treatment×length | 0.0184 | | 0.297 | |  |
|  | Boldness×length | 0.0005 | | 0.481 | |  |
|  | Treatment | 0.7491 | | 0.125 | |  |
|  | Boldness | -0.0002 | | 0.388 | |  |
|  | Length | 0.0271 | | 0.054 | |  |
| Individual social differentiation | Treatment×boldness×length | -0.0002 | | 0.172 | |  |
|  | Treatment×boldness | -0.0009 | | 0.157 | |  |
|  | Treatment×length | 0.0207 | | 0.131 | |  |
|  | Boldness×length | 0.0009 | | 0.488 | |  |
|  | Treatment | -0.2280 | | 0.380 | |  |
|  | Boldness | 0.0008 | | 0.331 | |  |
|  | Length | -0.0061 | | 0.148 | |  |
| Individual group size change | Treatment×boldness×length | <0.0001 | | 0.535 | |  |
|  | Treatment× length | 0.0190 | | 0.525 | |  |
|  | Treatment×boldness | 0.0013 | | 0.722 | |  |
|  | Boldness×length | 0.0004 | | 0.487 | |  |
|  | Length | 0.0044 | | 0.336 | |  |
|  | Boldness | 0.0002 | | **0.017** | |  |
|  | Treatment | **0.9776** | | **<0.001** | |  |
| Individual social differentiation change | Treatment×boldness×length | <0.0001 | | 0.122 | |  |
|  | Boldness×length | 0.0003 | | 0.381 | |  |
|  | Treatment×length | 0.0124 | | 0.230 | |  |
|  | Treatment×boldness | -0.0022 | | 0.181 | |  |
|  | Boldness | -0.0161 | | 0.478 | |  |
|  | Length | -0.0147 | | 0.426 | |  |
|  | Treatment | -0.2896 | | 0.062 | |  |
